# Retinoid and carotenoid status in serum and liver among patients at high-risk for liver cancer

**DOI:** 10.1186/s12876-016-0432-5

**Published:** 2016-02-29

**Authors:** Yachana Kataria, Ryan J. Deaton, Erika Enk, Ming Jin, Milita Petrauskaite, Linlin Dong, Joseph R. Goldenberg, Scott J. Cotler, Donald M. Jensen, Richard B. van Breemen, Peter H. Gann

**Affiliations:** Department of Laboratory Medicine, Boston Children’s Hospital, Boston, MA USA; Department of Pathology, University of Illinois at Chicago, Chicago, IL USA; Department of Hepatology, Loyola University, Chicago, IL USA; Center for Liver Diseases, University of Chicago, Chicago, IL USA; Department of Medicinal Chemistry & Pharmacognosy, University of Illinois at Chicago, Chicago, IL USA

**Keywords:** Vitamin A, HCV, Diet, Biomarker

## Abstract

**Background:**

Approximately 2.7 million Americans are chronically infected with hepatitis C virus (HCV). HCV patients with cirrhosis form the largest group of persons at high risk for hepatocellular carcinoma (HCC). Increased oxidative stress is regarded as a major mechanism of HCV-related liver disease progression. Deficiencies in retinoid and carotenoid antioxidants may represent a major modifiable risk factor for disease progression. This study aims to identify key predictors of serum antioxidant levels in patients with HCV, to examine the relationship between retinoid/carotenoid concentrations in serum and hepatic tissue, to quantify the association between systemic measures of oxidative stress and antioxidant status, and to examine the relationship between retinoids and stellate cell activation.

**Methods:**

Patients undergoing liver biopsy (*n* = 69) provided fasting blood, fresh tissue, urine and completed a diet history questionnaire. Serum and questionnaire data from healthy volunteers (*n* = 11), normal liver tissue from public repositories and patients without liver disease (*n* = 11) were also collected. Urinary isoprostanes, serum and tissue retinoid concentrations were obtained by UHPLC-MS-MS. Immunohistochemistry for αSMA was performed on FFPE sections and subsequently quantified via digital image analysis. Associations between urinary isoprostanes, αSMA levels, and retinoids were assessed using Spearman correlation coefficients and non-parametric tests were utilized to test differences among disease severity groups.

**Results:**

There was a significant inverse association between serum retinol, lycopene, and RBP4 concentrations with fibrosis stage. Serum β-carotene and lycopene were strongly associated with their respective tissue concentrations. There was a weak downward trend of tissue retinyl palmitate with increasing fibrosis stage. Tissue retinyl palmitate was inversely and significantly correlated with hepatic αSMA expression, a marker for hepatic stellate cell activation (*r* = −0.31, *P* < 0.02). Urinary isoprostanes levels were inversely correlated with serum retinol, β-carotene, and RBP4.

**Conclusions:**

A decrease in serum retinol, β-carotene, and RBP4 is associated with early stage HCV. Retinoid and carotenoid levels decline as disease progresses, and our data suggest that this decline occurs early in the disease process, even before fibrosis is apparent. Measures of oxidative stress are associated with fibrosis stage and concurrent antioxidant depletion. Vitamin A loss is accompanied by stellate cell activation in hepatic tissue.

**Electronic supplementary material:**

The online version of this article (doi:10.1186/s12876-016-0432-5) contains supplementary material, which is available to authorized users.

## Background

The progression of hepatitis C virus (HCV) infection can lead to cirrhosis and in some cases hepatocellular carcinoma (HCC), which has limited treatment options and poor prognosis. Approximately 2.7 million Americans are chronically infected with HCV. HCV patients with cirrhosis form the largest group of persons at high risk for HCC [1–5]. Oxidative stress, resulting from chronic inflammation, is purported to be a major mechanism for hepatic fibrosis and cirrhosis [6]. An imbalance between production of reactive oxygen species and antioxidant defense induces a number of pathophysiological changes in the liver, including activation of hepatic stellate cells (HSCs), oxidative damage to lipids, nucleotides and proteins, and initiation of proliferative processes associated with regeneration. Vitamin A and its carotenoid precursors are an important part of the body’s antioxidant defense, due to their ability to scavenge and directly neutralize free radicals in the tissue. In normal liver, quiescent HSCs are responsible for the storage of more than 90 % of the body’s vitamin A reserves as retinyl esters. Upon chronic liver injury HSC undergo activation, lose their capacity to store vitamin A while acquiring contractile, proliferative and proinflammatory properties that are believed to play a major role in fibrogenesis [7]. Hepatic αSMA expression in the parenchyma is a marker of activated HSCs due to their myofibroblast phenotype. However, it is uncertain if retinoid loss is required for stellate cell activation and if retinoid loss accelerates activation.

In this context, deficiencies in dietary antioxidants, such as retinoids and carotenoids, could represent a major modifiable risk factor for chronic liver disease (CLD) progression. Two prospective epidemiological studies evaluating the relationship between serum retinoids and liver cancer among subjects with chronic hepatitis B found that higher pre-diagnostic serum retinol was strongly associated with a subsequent reduced risk of liver cancer [8, 9]. More recently, a large cohort study in Finland observed that higher baseline serum retinol and β-carotene were inversely associated, many years later, with the incidence of liver cancer and death from CLD [10]. Additionally, a randomized trial has reported compelling evidence that polyprenoic acid, a synthetic retinoid, reduced the incidence of second primary liver tumors and prolonged survival in HCC patients [11]. A small number of studies to date have found that these serum micronutrients are depleted in cirrhotic patients and only three studies to date have assessed hepatic antioxidant levels in small and generally heterogeneous populations of pre-cirrhotic individuals [12–19].

Despite vast improvements in HCV treatment in recent years, there are potential opportunities for therapeutic or preventive strategies involving antioxidant repletion, particularly in patients who have progressed to cirrhosis even if they achieve virologic cure [20–22]. These limitations highlight a crucial need to develop adjuvant strategies to prevent progression in high-risk individuals [23]. However, supplementation with vitamin A itself must be approached cautiously in individuals with liver disease, as hypervitaminosis A causes accelerated liver fibrosis and may also promote carcinogenesis [24]. Therefore, there is a need for more information on the spectrum and causes of retinoid and carotenoid depletion in the HCV-infected population, so that optimal strategies for clinical trials can be identified.

Among HCV-infected persons, antioxidant depletion could be explained by a combination of dietary, lifestyle, and physiological factors. Apart from processes directly linked to HCV infection, these factors include inadequate dietary antioxidant intake, smoking, alcohol intake or diabetes, all of which have been reported to diminish defense against oxidative stress. The present study aims to: a) determine the prevalence and predictors of retinoid and carotenoid depletion in a well-defined patient population, b) examine the relationships between retinoid and carotenoid concentrations in serum and hepatic tissue, c) quantify the association between systemic measures of oxidative stress and antioxidant status, and d) examine the relationship between antioxidant levels and stellate cell activation. We postulated that lower retinoid and carotenoid concentrations and higher levels of oxidative stress would be associated with fibrosis stage among HCV-infected patients.

## Methods

### Study population

We conducted a cross-sectional study among patients with HCV at the University of Illinois at Chicago (UIC) and University of Chicago (UC). A total of 91 subjects were included in this study; however not every subject fulfilled every component of the study. We consented and consecutively enrolled patients with confirmed HCV undergoing percutaneous or transvascular liver biopsy (*n* = 69) who provided fasting blood, fresh tissue, urine, and completed a diet history questionnaire. At the time of the biopsy, liver histology was staged into F0-4 according to the Batts-Ludwig scoring system. Subjects with F0 were categorized as having no fibrosis. Subjects with fibrosis stage 1–2 and fibrosis 3–4 were categorized as mild/moderate and severe fibrosis, respectively. The liver histology staging criteria was utilized to define fibrosis stage in our analysis.

All biopsies were performed with 16 or 18-gauge needles; an average of 7.7 mm^3^ fresh tissue was taken from the end of each core and snap frozen for research purposes. We collected serum, urine, and questionnaire data from healthy volunteers (*n* = 11). We obtained post-mortem normal liver tissue from the Cooperative Human Tissue Network (CHTN) repository (*n* = 8); there was no corresponding serum, urine and questionnaire data for these individuals. The remaining controls included patients with no chronic liver disease (*n* = 3) who provided serum, tissue, urine, and questionnaire data. The UIC and UC Institutional Review Boards approved the study. An informed consent to participate in the study was obtained from participants.

### Dietary assessment

The National Cancer Institute Diet History Questionnaire I (DHQ) was completed by all participants who provided serum. The DHQ was subsequently analyzed by the Diet*Calc Analysis Program (Version 1.4.3. National Cancer Institute, Applied Research Program). This program generated nutrient estimates based on frequency and portion sizes over the past year. Approximate retinoid and carotenoid intake was calculated from the DHQ. Pre-formed vitamin A is primarily found in animal sources such as eggs, dairy produce, fish, and meat. Carotenoids, which are found primarily in plant foods, include α-carotene, β-carotene, β-cryptoxanthin, lutein, and zeaxanthin; of those only α-carotene, and β-carotene are pro-vitamin A carotenoids; i.e., vitamin A precursors. To account for both pre-formed and pro-vitamin A dietary sources, the recommended dietary allowance for vitamin A is expressed in retinol activity equivalents (RAE).

### Serological measurements

Serum analyses were performed by the clinical pathology laboratories at UIC. The serum chemistry panel included liver function tests aspartate aminotransferase (AST), alanine aminotransferase (ALT), international normalized ratio (INR), albumin, total bilirubin, total protein, BUN, hemoglobin, creatinine, and platelet count. Fasting serum insulin, glucose, and high sensitivity C-reactive protein, a marker of systemic inflammation (hs-CRP) were measured in single batches on SYNCHRON® Systems from Beckman Coulter (Pasadena, California). Based on blinded duplicates from a quality control pool of serum, intra-batch coefficients of variation (CV) aliquots for fasting serum insulin, glucose, and hs-CRP were 2.4, 0.65, and 0.98 %, respectively. Insulin resistance was measured using the homeostatic model assessment of insulin resistance (HOMA-IR). Predictive markers of fibrosis, aminotransferase to platelet ratio index (APRI) and Fibrosis 4 (FIB4) were calculated using published formulas [25, 26].

### Serum retinoid/carotenoid assays

Singlicate measurements of retinoids and carotenoids in fasting serum samples were performed by using atmospheric pressure chemical ionization mass spectrometry as described previously, with the following changes [27]. Ultrahigh pressure liquid chromatography-tandem mass spectrometry (UHPLC-MS/MS) was carried out using a Shimadzu (Kyoto, Japan) LCMS-8040 triple quadrupole mass spectrometer equipped with a Shimadzu Shim-pack XR-ODSIII column (2.0 × 50 mm, 1.6 μm) at 35 °C. After holding at 5:95 (v/v) methyl-*tert*-butyl ether/methanol for 0.3 min, a 0.45-min linear gradient was used from 5 to 30 % methyl-*tert*-butyl ether at a flow rate of 0.6 mL/min. Carotenoids and retinoids were detected by MS/MS during the same analysis using polarity switching and the following selected reaction monitoring transitions: lycopene *m/z* 536 to 467 (-), [^13^C_10_]-lycopene (internal standard) *m/z* 546 to 477 (-), β-carotene *m/z* 536 to 536 (-), lutein *m/z* 551 to 135 (+), retinol and retinyl palmitate *m/z* 269 to 93 (+).

Based on blinded replicates from a quality control pool of serum, mean intra- and inter- batch CVs for retinol in serum samples were 9.3 and 13.9 %, respectively. Average intra- and inter- batch CV for all serum retinoids and carotenoids were 13.9 and 9.7 %, respectively.

The Relative Dose Response (RDR) test has been proposed as a better alternative for determining vitamin A deficiency compared to serum retinol. A retinol increase of greater than 20 % following a challenge dose of retinyl palmitate is considered a positive test indicating deficient liver reserves. Serum delta RDR values were calculated according to the following formula: $100\times \frac{A_5-{A}_0}{A_5}$. A_0_ is the serum retinol concentration at baseline, and A_5_ is the serum retinol concentration at 5 h post-retinol dose. Test participants provided serum for baseline retinol levels after an overnight fast, and then ingested 1000 RAE of retinyl palmitate dissolved in corn oil on a cracker. Serum retinol levels were measured again five hours later. Of the 24 subjects who completed the RDR, eleven had tissue available.

### RBP4 measurement

Serum retinol binding protein 4 (RBP4) was measured in duplicate using an enzyme-linked immunosorbent assay (ELISA) (ALPCO Diagnostics, Salem, NH) according to the manufacturer’s instructions. The kit utilized a polyclonal rabbit anti-RBP antibody.

### Tissue retinoid/carotenoid assays

Hepatic tissue retinoid and carotenoid measurements were performed using UHPLC-MS/MS as described above, except that the tissue (0.5 to 5 mg) was homogenized in 200 μl water and extracted twice using 600 μl portions of ethanol/hexane (20:80; v/v) containing 0.1 % butylated hydroxytoluene. The same protocol was used for all of the hepatic tissue available. Based on adjacent samples from a random subject within each batch, mean intra-batch CV for hepatic tissue retinyl palmitate, β-carotene, and lycopene were 6.9, 10.0 and 4.1 %, respectively.

### Urinary isoprostanes

Urine concentrations of 8-iso-PGF2α, a marker of lipid peroxidation were measured using a rapid UHPLC-MS/MS assay as described previously [28]. Based on anonymous replicates from a quality control pool of urine, mean intra- and inter-batch CV for the urinary isoprostanes measurements were 6.4 and 7.8 %, respectively.

### Immunohistochemistry

Tissue sections of 4 μm each were placed on charged slides, dehydrated, and then deparaffinized. Immunostaining was carried out using a BondRX (Lecia Biosystems, Buffalo Grove, IL) autostainer with a mouse monoclonal antibody for αSMA (DAKO, Clone 1A4) in a 1:2000 dilution for 15 min at room temperature to identify HSC. The sections were then incubated in rabbit anti-mouse IgG (Bond Polymer Refine Detection, Leica Biosystems) for 20 min. No antigen retrieval was used for this stain. Color reaction was developed using diaminobenzidine (DAB) as the chromagen, followed by counterstaining with hematoxylin. Positive and negative controls were included in each batch.

Slides were scanned at 20× on an Aperio ScanScope® CS whole-slide digital microscope (Leica Biosystems). A digital draw tool was used to identify hepatic parenchymal areas. Large vessels, inflammation, and artifacts (e.g.*,* folds, debris, etc.) were excluded from analysis. αSMA quantitation was restricted to the hepatic parenchymal region to exclude αSMA positive cells (i.e., portal fibroblasts and bone marrow derived collagen-producing cells) in the portal region [29]. Definiens Tissue Studio® 3.6.1 (Definiens, Munich, Germany), a digital image analysis platform, was used to measure the percent positivity of αSMA stain area within the hepatic parenchyma.

### Statistical analysis

Frequency distributions of dietary intake, urinary isoprostanes, retinoid, and carotenoid concentrations were examined for normality. Scatterplots and Spearman rank correlation coefficients were used to examine relationships among the variables of interest. A *P*-value of < 0.05 was considered statistically significant, and all tests were two-sided. Analyses were performed using SAS Version 9.2 (SAS, Inc., Cary, NC).

## Results

Table 1 shows selected demographics and clinical characteristics of the study participants at both institutions by fibrosis stage. Participants in the control group were more likely to be white, overweight, and non-smokers. There were no differences in ethnicity, BMI, smoking status, or diabetes among the disease groups. The no fibrosis group was more likely to be overweight or obese compared to the mild/moderate fibrosis group (94 % vs 59 %). A majority of the HCV-positive subjects had either genotype 1a (52 %) or 1b (38 %) as reported by medical records. The median AST, APRI, and FIB-4 levels in disease subjects were positively associated with the fibrosis stage (Additional file 1: Table S1). However, hs-CRP concentrations were inversely and significantly associated with fibrosis stage (*P* < 0.05) (Additional file 1: Table S1).

**Table 1:** Selected characteristics of the study population by fibrosis stage

Characteristics	Control	No fibrosis	Mild/Moderate fibrosis	Severe fibrosis	Total	*P*-value*	*P*-value**
	*n* = 22	*n* = 18	*n* = 34	*n* = 17	*n* = 91		
Age - Median (IQR)	45 (42–54)	55 (51–62)	53 (48–58)	56 (53–57)	53 (46–58)	0.17	0.17
Gender							
Male	12	6	18	10	46	0.42	0.27
Female	10	12	16	7	45		
Ethnicity							
Asian	0	0	1	0	1	**<0.01**	0.68
Black	2	12	17	7	38		
White	18	6	15	9	48		
Unknown	2	0	1	1	4		
BMI							
< 25	0	1	13	4	18	**0.02**	0.11
25–30	5	9	12	7	33		
> 30	9	8	8	6	31		
Unknown	8	0	1	1	10		
Smoking Status							
Never	17	7	10	7	41	**0.02**	0.60
Former	2	7	11	7	27		
Current	3	4	13	3	23		
Diabetes							
Yes	3	2	3	4	12	0.56	0.33
No	19	16	31	13	79		
HCV Genotype							
1	–	11	29	13	53	0.15	0.15
2	–	1	0	0	1		
3	–	0	0	1	1		
Unknown	–	6	5	3	14		

*P-values are Fisher exact (2-tailed) for comparison of proportions and Kruskal-Wallis test for comparison of medians amongst all groups

**P-values are Fisher exact (2-tailed) for comparison of proportions and Kruskal-Wallis test for comparison of medians amongst diseased groups

Bold face represents statistically significant values

**Fig. 1:**
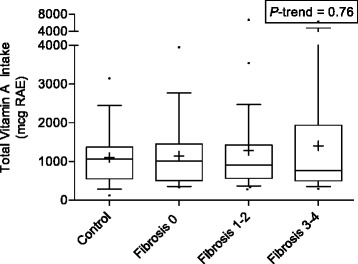
Boxplots (Whiskers = 10th and 90th percentile) of total vitamin A intake (mcg retinol activity equivalents) by fibrosis stage

Dietary intake of individual retinoids and carotenoids did not differ between controls and HCV subjects. There was evidence of a downward trend for total vitamin A intake (expressed as retinol activity equivalents, RAE) with increasing fibrosis stage, however this trend was weak and not readily distinguishable from chance, as shown in Fig. 1. Total vitamin A, or individual retinoid and carotenoid intake also did not differ by fibrosis stage or predictive markers of fibrosis (APRI and FIB-4). Mean total vitamin A intake was 1182 and 1295 mcg RAE for men and women, respectively; values well above the general population based on data from NHANES III (682 and 606 mcg RAE, for men and women, respectively). Total vitamin A or individual retinoid and carotenoid intake levels were not associated with BMI, smoking status, alcohol consumption, or insulin resistance.

**Fig. 2:**
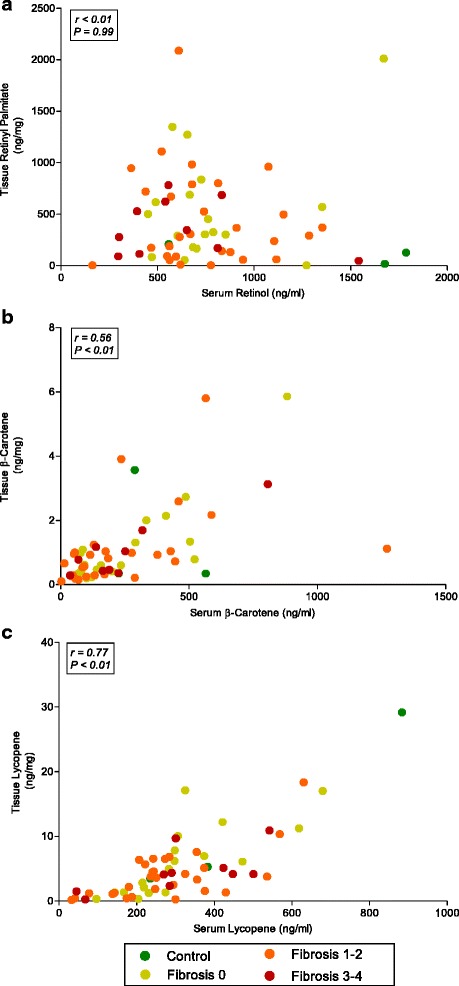
Scatterplots showing correlations (Spearman r) between **a** Serum retinol and tissue retinyl palmitate (*n* = 60), **b** Serum and tissue β-carotene (*n* = 58), and **c** Serum and tissue lycopene (*n* = 60)

Dietary β-carotene and lutein intake were positively and significantly correlated with their respective serum concentrations (β-carotene: *r* = 0.24, *P =* 0.05; lutein: *r* = 0.33, *P <* 0.01). However, total vitamin A intake did not correlate with serum retinol concentrations (*r* = 0.07, *P =* 0.53). Similarly, no relationships between intake and serum were observed for other dietary carotenoids. Tissue retinoid and carotenoid levels were not associated with dietary intake (data not shown). Figure 2 shows that serum retinol and hepatic retinyl palmitate concentrations, the respective dominant forms of vitamin A in serum and liver, were not correlated (*r* < 0.01, *P =* 0.99). However, serum and tissue concentrations of β-carotene (*r* = 0.56, *P <* 0.01) and lycopene (*r* = 0.77, *P <* 0.01) were moderately correlated in this study population.

**Fig. 3:**
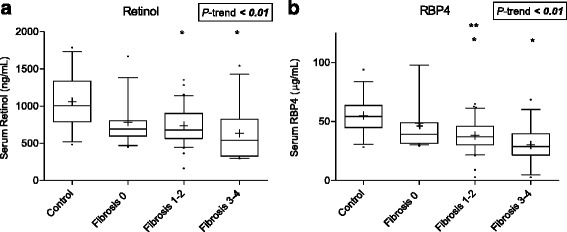
Boxplots (Whiskers = 10th and 90th percentile) for serum concentrations of **a** retinol and **b** RBP4 by fibrosis stage. *P < 0.05 compared to Control **P < 0.05 compared to Fibrosis 0 group

**Fig. 4:**
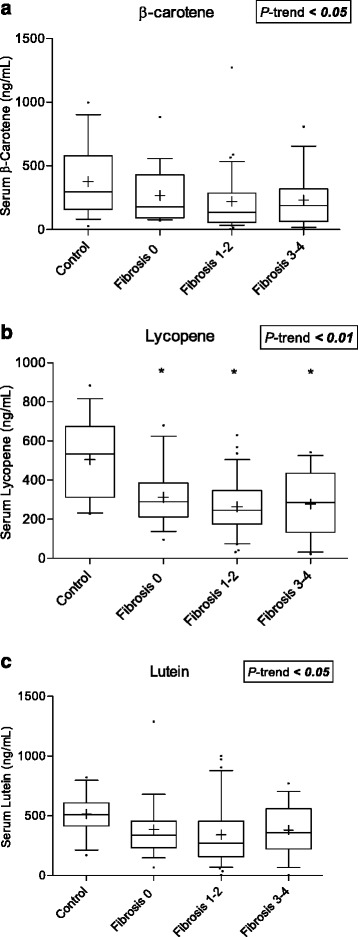
Boxplots (Whiskers = 10th and 90th percentile) for serum concentrations of **a** β-carotene, **b** lycopene, and **c** lutein by fibrosis stage. *P < 0.05 compared to control group

Serum retinol and RBP4 concentrations were significantly lower in HCV subjects compared to controls, although no HCV subjects met the standard serum criteria for vitamin A deficiency (<200 ng/mL). Figure 3 shows a significant downward trend of serum retinol and RBP4 concentrations with increasing hepatic fibrosis. Similar relationships were also observed for serum β-carotene, lycopene, and lutein concentrations (Fig. 4). No relationships were observed for serum retinoids and carotenoids with either APRI or FIB-4 scores (data not shown). However, RBP4 levels were inversely and significantly correlated with AST and ALT levels (*r* = −0.34, *P <* 0.01; *r* = −0.28, *P* = 0.015, respectively). Individual retinoid and carotenoid concentrations did not vary according to sex, BMI, smoking status, alcohol consumption, or insulin resistance. Of the 24 HCV participants who completed the RDR test, two of them had RDR values >20 %, suggestive of a vitamin A deficiency. There was no apparent relationship between delta RDR retinol concentrations and retinyl palmitate concentrations in hepatic tissue (Spearman *r* = −0.03, *P* = 0.92) or fibrosis stage (data not shown).

**Fig. 5:**
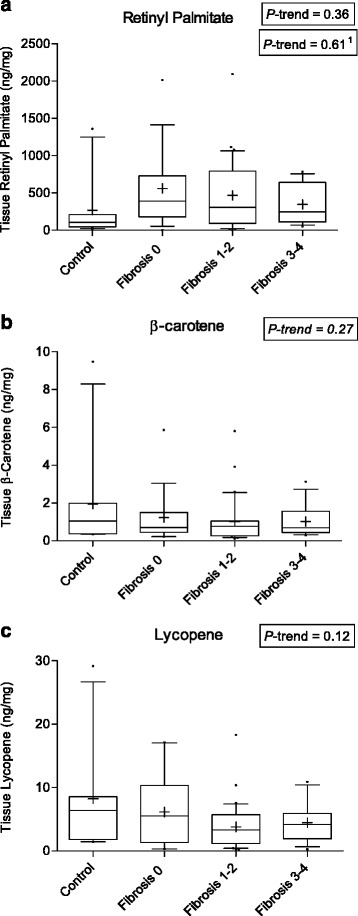
Boxplots (Whiskers = 10th and 90th percentile) for tissue concentrations of **a** retinyl palmitate, **b** β-carotene, and **c** lycopene by fibrosis stage. ^1^P-trend excludes control group

Hepatic retinyl palmitate levels were lower in control tissue samples, which were mostly obtained post-mortem, compared to diseased subjects (Wilcoxon *P* = 0.04). In contrast, hepatic lycopene and β-carotene concentrations were higher in controls compared to diseased subjects (Wilcoxon *P* = 0.07, *P* = 0.34, respectively). There was a weak inverse relationship between hepatic retinyl palmitate concentration and fibrosis stage among all subjects (*P* = 0.36*)* (Fig. 5). Furthermore, hepatic β-carotene and lycopene concentrations showed a weak downward trend with increasing fibrosis stage. Hepatic retinyl palmitate was positively and significantly correlated with APRI, FIB-4, ALT, and AST (*r* = 0.27, *P =* 0.03; *r* = 0.29 *P =* 0.02; *r* = 0.30, *P =* 0.015; and *r* = 0.24, *P =* 0.05, respectively). These relationships were not observed for tissue carotenoids.

**Fig. 6:**
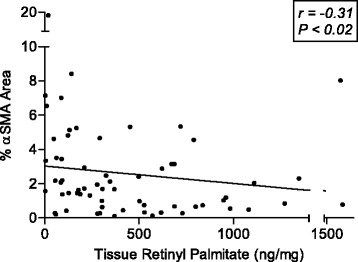
Scatterplots showing correlations (Spearman r) between tissue retinyl palmitate and % marker area of αSMA protein expression (*n* = 65)

Parenchymal αSMA expression in hepatic tissue appeared to increase only among subjects with fibrosis 3–4 (Additional file 2: Figure S1) (Wilcoxon *P* = 0.12). αSMA expression was not associated with serum retinol concentrations (*r* = −0.03, *P =* 0.79). However, αSMA expression was inversely and significantly correlated with tissue retinyl palmitate concentrations (*r* = −0.31, *P =* 0.013) (Fig. 6). This relationship was not observed for any other tissue carotenoids (data not shown). In particular, hepatic lycopene levels were not correlated with αSMA expression (*r* = −0.03, *P =* 0.81).

Urinary isoprostane levels were positively and significantly associated with fibrosis stage (Additional file 3: Figure S2). Serum retinol, β-carotene, and RBP4 concentrations were all inversely and significantly associated with urinary isoprostane concentrations. Tissue retinoid concentrations were not correlated with urinary isoprostane levels (Table 2). However, both serum and hepatic lycopene were suggestively correlated (*r* = −0.18, *P =* 0.12; *r* = -0.22, *P =* 0.09, respectively).

**Table 2:** Spearman correlations between urinary isoprostanes, serum and tissue retinoids/carotenoids

	Urinary isoprostanes (ng/mg creatinine)	
Serum Retinoids/Carotenoids (ng/mL) (*n* = 77)	r	*P*-value
Retinol	**−0.23**	**0.05**
Lycopene	−0.18	0.12
β-Carotene	**−0.22**	**0.05**
Lutein	−0.12	0.31
RBP4 (ug/L)	**−0.25**	**0.03**
Tissue Retinoids/Carotenoids (ng/mg) (*n* = 60)		
Retinol	−0.05	0.72
Lycopene	−0.22	0.09
β-Carotene	−0.08	0.53
Retinyl Palmitate	−0.03	0.79

Bold face represents statistically significant values

## Discussion

Our results confirm that depletion of vitamin A, lycopene, and β-carotene is widespread among patients with chronic HCV infection. This phenomenon seems to occur early in the disease process, even before fibrosis is apparent, and cannot be explained, based on our results, by diet, obesity, alcohol intake, smoking, or insulin resistance. Inverse associations with fibrosis progression were more apparent for serum as opposed to hepatic levels of retinoids and carotenoids, and were especially clear for serum retinol and RBP4. While we found relatively strong correlations between serum and liver tissue for lycopene and β-carotene, hepatic retinyl palmitate was poorly correlated with serum retinol, suggesting differential factors modulating these levels [17, 18]. It is also possible that declines in serum retinoids appear earlier in the disease process than declines in hepatic stores. We further observed that depletion of serum antioxidants is linked to increasing levels of urinary isoprostanes, which are reflective of systemic oxidative stress due to lipid peroxidation. An important finding was that hepatic retinyl palmitate levels were significantly and inversely associated with stellate cell activation, as measured by αSMA expression in liver biopsy specimens. Taken together, results from this cross-sectional analysis support the hypotheses that depletion of retinoid and carotenoid antioxidants occurs early in the disease process and that this depletion parallels an increase in oxidative stress and evidence of hepatic stellate cell activation.

In the present study, the reduced serum retinol levels associated with CLD progression were well above the widely accepted WHO cut-off point of 200 ng/mL for vitamin A deficiency. Moreover, only two of the HCV participants had a positive RDR test, indicating inadequate liver vitamin A reserve. Serum retinol and β-carotene levels in our HCV participants were also generally comparable to the NHANES III participants, a nationally representative sample of the US population [30]. Reduced dietary intake of retinoids and carotenoids do not appear to be responsible for the observed associations with fibrosis stage, which could be the result of diminished storage capacity, increased metabolism or defective mobilization of retinol due to impaired RBP4 synthesis [15, 31, 32]. In any event, a cross-sectional study such as this is unable to determine if antioxidant depletion is a causal factor in fibrosis progression, or simply an epiphenomenon that accompanies progression.

The current study supports prior evidence that serum RBP4 concentrations are inversely related to disease severity in HCV patients [33, 34]. We observed a high correlation between serum RBP4 and retinol (*r* = 0.78, *P* < 0.001). Serum RBP4 measured by an enzyme immunoassay might be a feasible and cost-effective alternative for assessing vitamin A status. No association was detected between RBP4 levels and albumin, suggesting that generally decreased hepatic protein synthesis did not contribute to the reduction in RBP4. However, impaired mobilization of hepatic stores could explain the decrease of RBP4 seen in CLD. Increased serum RBP4 has been reported to contribute to insulin resistance associated with type 2 diabetes and obesity, which are possible risk factors for CLD progression [35]. We observed no correlation between RBP4 concentrations and BMI, glucose, or insulin levels. The possible effects of reduced RBP4 levels on insulin resistance among CLD patients might warrant further study.

A small number of studies have evaluated serum and tissue concentrations of dietary antioxidants in patients with CLD; although these have focused on more severe, later stage disease. The lack of correlation between serum retinol and tissue retinyl palmitate could be explained by impaired release of retinol from damaged hepatocytes or by the presence of homeostatic mechanisms that maintain hepatic retinol until vitamin A stores are severely depleted. We did not observe a definite decline in hepatic retinyl palmitate concentrations with increasing disease severity in earlier stages of CLD. Yadav et al. reported lower levels of retinyl esters in 20 HCV patients compared to controls [17]. In our study, however, the lower concentration in controls could have been caused by the degradation of retinyl palmitate into retinol in the cadaver tissue by endogenous esterase. Indeed, we observed higher levels of hepatic retinol in the control subjects (data not shown). It has been reported that patatin-like phospholipase domain-containing protein 3 (PNPLA3) regulates retinyl ester release. Specifically, carriers of the I148M mutation in PNPLA3 have intracellular hepatic retention of retinyl palmitate [36]. Subsequently, Mondul et al. found in NAFLD patients that carriers of the PNPLA3 I148M mutation have lower circulating levels of retinyl palmitate and RBP4 but not β-carotene concentrations [37]. PNPLA3 is also a strong determinant for HCC [38]. Taken together, it is possible that the intracellular retention of vitamin A is contributing to fibrosis progression and HCC. The observed reduction in serum Vitamin A levels may reflect a pseudo-deficiency. Future studies investigating the PNPLA3 genotype in this population may provide further insight. The reason for the positive relationship between hepatic retinyl palmitate and liver enzymes is unclear, but this relationship would also be consistent with mechanisms that favor retention of vitamin A in the presence of early but ongoing liver damage.

Lycopene cannot be converted to vitamin A and thus it presents an attractive potential alternative for antioxidant supplementation in CLD. Adverse effects have not been reported with consuming lycopene supplements or high amounts of lycopene-rich foods [39, 40]. We observed an inverse relationship of serum lycopene levels with fibrosis stage. Moreover, unlike vitamin A, there was also a strong correlation between serum and hepatic lycopene levels, thus indicating that dietary supplementation could result in higher hepatic lycopene concentrations. Yuan, et al. demonstrated an inverse association between lycopene concentration in baseline serum and risk of developing HCC in China. Lycopene is a potent carotenoid antioxidant that is also thought to affect processes related to mutagenesis, carcinogenesis, cell differentiation, and proliferation [41, 42]. Epidemiological data suggest that lycopene may act as a chemopreventive agent for many cancer types such as prostate, breast, and lung [43]. In vitro studies have observed that lycopene can inhibit mouse and human hepatocytes by inducing cell cycle arrest [44, 45]. However, dietary lycopene did not prevent liver cancer in an in vivo model of spontaneous hepatocarcinogenesis [46]. These discrepant results warrant further investigation.

Serum hs-CRP is synthesized by hepatocytes in response to inflammation and is regulated by pro-inflammatory cytokines such as IL-6. In a recent cohort analysis in China, higher serum CRP levels at baseline were associated with liver cancer incidence and death from CLD [47]. As such, we hypothesized that higher levels of hs-CRP would occur with increasing fibrosis stage. However, in this study, hs-CRP concentrations decreased with increasing fibrosis stage. While these findings are counterintuitive, a few smaller studies have suggested similar results [48–50]. Nasciemento, et al. observed lower hs-CRP to IL-6 ratio compared to controls, suggesting that IL-6 stimulation of CRP production in the liver might be mitigated by HCV [48]. It appears that CRP production is affected early during the natural history of HCV infection, thus negating its utility as a biomarker of inflammation in this population.

F_2_-isoprostanes are a sensitive and validated urinary marker of systemic oxidative stress due to lipid peroxidation. In our study, smokers had higher isoprostane levels compared to non-smokers. Our data indicate a positive association between urinary isoprostane concentrations and fibrosis stage. To our knowledge, this extends beyond previous data that have shown patients with CLD and cirrhosis have increased isoprostane levels compared to controls [16]. As hypothesized, we observed a significant inverse association of F_2_-isoprostanes with serum retinol, β-carotene, and RBP4 levels. However, tissue retinoid and carotenoid levels were not associated with urinary isoprostane levels with the possible exception of lycopene. Urinary isoprostanes are a measure of total body lipid peroxidation, and therefore do not reliably reflect oxidative stress in the target organ.

As we expected, subjects with moderate to severe fibrosis had higher levels of αSMA expression in hepatic tissue compared to subjects with mild fibrosis or none at all. Thus, it appears that HSC activation might not be apparent, at least by immunohistochemical technique, until substantial fibrosis is present. Our data indicate that vitamin A depletion could occur prior to development of significant fibrosis and evidence of HSC activation. The hepatic vitamin A depletion might be attributable to either loss of storage capacity or increased consumption via autophagy. Increased metabolism of vitamin A droplets through autophagic mechanism generates substrates needed for energy intensive pathways to meet the metabolic demands of proliferation, fibrogenesis, and contractility [51].

This is the first comprehensive analysis to examine the interrelationships among dietary, serum, and hepatic levels of retinoids and carotenoids with early progression of disease in HCV patients. The present study, to our knowledge, is the largest to date to quantitate hepatic retinoids and carotenoids. In addition, the study benefitted from quantitative image analysis that provided objective and reproducible measures of αSMA expression in liver biopsy specimens. However, certain limitations are acknowledged including, a limited sample size for specific subgroups and a cross sectional design that cannot be used to infer causal direction. Unfortunately, we were unable to administer the RDR to more patients due to the additional blood sample required five hours after the initial retinyl ester dose. Additionally, the post-mortem tissue samples of normal liver were not ideal as they could have undergone artifactual changes in retinoid and carotenoid concentrations.

Findings from this study could be applicable to the emerging epidemic of obesity-related non-alcoholic fatty liver disease (NAFLD) and non-alcoholic steatohepatitis (NASH) [52]. Oxidative stress is believed to play a key role in these disease processes, similar to HCV-related liver disease. Currently, there are no specific therapies for NAFLD beyond dietary modifications and exercise. Lower levels of retinoids have been implicated in NAFLD. Transgenic mice with impaired retinoid signaling develop steatohepatits and eventually HCC [23]. It has also been found that NAFLD patients have higher levels of oxidative stress and lower vitamin A intake, independent of metabolic syndrome status, suggesting that adequate vitamin A intake is important in protecting against oxidative stress in NAFLD patients [53].

## Conclusions

 In conclusion, a decrease in serum retinol, β-carotene, and RBP4 is associated with early stage HCV. Retinoid and carotenoid levels decline as disease progresses, and our data suggest that this decline occurs early in the disease process, even before fibrosis is apparent. Measures of oxidative stress are associated with fibrosis stage and concurrent antioxidant depletion. Vitamin A loss is accompanied by stellate cell activation in hepatic tissue. Our data highlight the potential importance of dietary retinoids and carotenoids as determinants of progression in early chronic liver disease and support the need for mechanistic studies leading to well-designed intervention studies.

## Additional files

Additional file 1: Table S1. Clinical assay results for the study population by fibrosis stage - median (interquartile range). (DOCX 38 kb) Additional file 2: Figure S1. Boxplots (10th and 90th Percentile) of percent protein expression of αSMA area by fibrosis severity. (EPS 108 kb) Additional file 3: Figure S2. Boxplots (10th and 90th Percentile) of urinary isoprostanes by fibrosis stage. (EPS 102 kb)

## Abbreviations

ALT: alanine aminotransferase
APRI: aminotransferase to platelet ratio index
AST: aspartate aminotransferase
CLD: chronic liver disease
FFPE: fresh frozen paraffin embedded
FIB-4: fibrosis-4
HCC: hepatocellular carcinoma
HCV: hepatitis C
hs-CRP: high sensitivity C-reactive protein
HSCs: hepatic stellate cells
NAFLD: non-alcoholic fatty liver disease
NASH: non-alcoholic steatohepatitis
RAE: retinol activity equivalents
RBP4: retinol binding protein 4
UHPLC-MS/MS: ultrahigh pressure liquid chromatography-tandem mass spectrometry
αSMA: α-smooth muscle actin
