# Supplementary material for: Retinoid and carotenoid status in serum and liver among patients at high-risk for liver cancer
Source: BMC Gastroenterol. 2016 Feb 29;16:30. doi: 10.1186/s12876-016-0432-5 (PMC4772305; doi:10.1186/s12876-016-0432-5)
Supplement: Additional file 1: Table S1. — Clinical assay results for the study population by fibrosis stage - median (interquartile range). (DOCX 38 kb) [file 12876_2016_432_MOESM1_ESM.docx]

| **Table S1. Clinical assay results for the study population by fibrosis stage - median (interquartile range)** | | | | | | |
| --- | --- | --- | --- | --- | --- | --- |
| **Laboratory Data** | **Control** | **No Fibrosis** | **Mild/Moderate Fibrosis** | **Severe Fibrosis** | ***P* - value**¹ | ***P* - value²** |
|  | n=14 | n=17 | n=34 | n=17 |  |  |
| **AST** (IU/L) | 23.5 (20.0-29.0) | 42.0 (30.0-60.0) | 46.5 (35.0-68.0) | 94.0 (43.0-127.0) | **<0.01** | **<0.02** |
| **ALT** (IU/L) | 22.5 (18.0-29.0) | 57.0 (31.0-67.0) | 56.5 (32.0-76.0) | 89.0 (42.0-115.0) | **<0.01** | 0.27 |
| **INR** | 1.10 (1.07-1.12) | 1.00 (0.95-1.1) | 1.02 (1.0-1.1) | 1.05 (1.0-1.1) | 0.08 | 0.08 |
| **Albumin** (g/dL) | 4.0 (3.9-4.3) | 4.1 (3.8-4.2) | 4.0 (3.8-4.2) | 4.0 (3.7-4.2) | 0.83 | 0.70 |
| **Total Bilirubin** (mg/dL) | 0.7 (0.5-0.9) | 0.6 (0.3-0.9) | 0.6 (0.5-0.8) | 0.8 (0.5-1.0) | 0.35 | 0.21 |
| **Total Protein** (g/dL) | 6.9 (6.6-7.2) | 7.6 (7.0-8.1) | 7.4 (7-8.1) | 7.2 (6.9-7.7) | **0.04** | 0.52 |
| **BUN** (mg/dL) | 12.5 (10.0-16.0) | 13.5 (11.0-16.0) | 12.0 (9.0-14.0) | 14.0 (11.0-15.0) | 0.26 | 0.14 |
| **Hemoglobin** (g/dL) | 14.2 (13.8-14.6) | 14.2 (12.8-14.8) | 14.4 (11.9-14.9) | 14.0 (13.5-15.2) | 0.78 | 0.99 |
| **Platelet Count** (10^9^/L) | 288 (238-292) | 207 (141-251) | 212 (181-258) | 190 (153-228) | 0.10 | 0.35 |
| **APRI^3^** | 0.18 (0.16-0.29) | 0.50 (0.42-0.83) | 0.51 (0.39-0.87) | 1.01 (0.48-2.03) | **<0.01** | **<0.05** |
| **FIB-4**^4^ | 0.7 (0.5-0.7) | 1.7 (1.2-2.2) | 1.5 (1.1-2.2) | 2.7 (1.7-3.6) | **<0.01** | **<0.02** |
| **Fasting Serum Assays** | n=14 | n=18 | n=32 | n=13 |  |  |
| **HS-CRP** (mg/L) | 2.3 (1.1-9.5) | 2.3 (0.6-2.1) | 0.7 (0.2-1.7) | 0.6 (0.5-2.1) | **0.04** | 0.39 |
| **Insulin** (µIU/mL) | 6.5 (5.3-14.5) | 9.4 (6.4-16.2) | 8.4 (6.4-15.9) | 11.8 (8.8-16.4) | 0.40 | 0.41 |
| **Glucose** (mg/dL) | 96 (81-120) | 89.5 (82-97) | 93 (182-98) | 94 (87-105) | 0.60 | 0.71 |
| **HOMA-IR Score** | 1.6 (1.1-3.6) | 2.3 (1.4-3.4) | 2.0 (1.2-4.0) | 2.7 (1.8-4.5) | 0.49 | 0.42 |
| ^1^ Kruskal-Wallis test for comparison of medians amongst all groups | | | | | |  |
| ^2^ Kruskal-Wallis test for comparison of medians amongst diseased groups | | | | | |  |
| ^3^ AST to Platelet Ratio Index (APRI) | | | | | |  |
| ^4^ Fibrosis 4 (FIB-4) is based on age, aspartate aminotransferase, alanine aminotransferase levels, and platelet counts | | | | | |  |
